# Morphometry Predicts Early GFR Change in Primary Proteinuric Glomerulopathies: A Longitudinal Cohort Study Using Generalized Estimating Equations

**DOI:** 10.1371/journal.pone.0157148

**Published:** 2016-06-10

**Authors:** Kevin V. Lemley, Serena M. Bagnasco, Cynthia C. Nast, Laura Barisoni, Catherine M. Conway, Stephen M. Hewitt, Peter X. K. Song

**Affiliations:** 1 Department of Pediatrics, Division of Pediatric Nephrology, University of Southern California Keck School of Medicine, Los Angeles, California, United States of America; 2 Department of Pathology, The Johns Hopkins School of Medicine, Baltimore, Maryland, United States of America; 3 Department of Pathology, Cedars-Sinai Medical Center, Los Angeles, California, United States of America; 4 Department of Pathology, University of Miami, Miami, Florida, United States of America; 5 National Cancer Institute, NIH, Bethesda, Maryland, United States of America; 6 Department of Biostatistics, University of Michigan, Ann Arbor, Michigan, United States of America; Emory University, UNITED STATES

## Abstract

**Objective:**

Most predictive models of kidney disease progression have not incorporated structural data. If structural variables have been used in models, they have generally been only semi-quantitative.

**Methods:**

We examined the predictive utility of quantitative structural parameters measured on the digital images of baseline kidney biopsies from the NEPTUNE study of primary proteinuric glomerulopathies. These variables were included in longitudinal statistical models predicting the change in estimated glomerular filtration rate (eGFR) over up to 55 months of follow-up.

**Results:**

The participants were fifty-six pediatric and adult subjects from the NEPTUNE longitudinal cohort study who had measurements made on their digital biopsy images; 25% were African-American, 70% were male and 39% were children; 25 had focal segmental glomerular sclerosis, 19 had minimal change disease, and 12 had membranous nephropathy. We considered four different sets of candidate predictors, each including four quantitative structural variables (for example, mean glomerular tuft area, cortical density of patent glomeruli and two of the principal components from the correlation matrix of six fractional cortical areas–interstitium, atrophic tubule, intact tubule, blood vessel, sclerotic glomerulus, and patent glomerulus) along with 13 potentially confounding demographic and clinical variables (such as race, age, diagnosis, and baseline eGFR, quantitative proteinuria and BMI). We used longitudinal linear models based on these 17 variables to predict the change in eGFR over up to 55 months. All 4 models had a leave-one-out cross-validated R^2^ of about 62%.

**Conclusions:**

Several combinations of quantitative structural variables were significantly and strongly associated with changes in eGFR. The structural variables were generally stronger than any of the confounding variables, other than baseline eGFR. Our findings suggest that quantitative assessment of diagnostic renal biopsies may play a role in estimating the baseline risk of succeeding loss of renal function in future clinical studies, and possibly in clinical practice.

## Introduction

Longitudinal statistical models predicting progression of kidney diseases are increasingly appreciated as instruments to guide treatment decisions or to identify high-risk individuals in clinical practice [1]. Most predictive models of kidney disease progression have either not included explicit structural parameters or included them only as qualitative or semi-quantitative variables. At the same time, some earlier studies have indicated the utility of renal structural parameters in predicting patient outcomes.

For example, morphometric assessment of cortical interstitial volume both correlated cross-sectionally with various aspects of renal function and predicted 10-year renal functional outcomes in several glomerular disorders [2]. Fractional interstitial area (an unbiased estimator of cortical interstitial volume fraction) and average glomerular tuft volume–both measured in baseline diagnostic biopsies–were among the five significant predictor variables in a study of loss of measured GFR over 4 to 5 years in IgA nephropathy [3]. The other significant variables in this study included the percentage of globally sclerotic glomeruli, serum creatinine and measured renal plasma flow. Finally, the cortical density of glomeruli has been reported to predict loss of GFR in different glomerular disorders [4, 5]. In a study of 65 patients with idiopathic membranous nephropathy and an estimated GFR (eGFR) ≥ 60 mL/min/1.73 m^2^ at baseline, of nine clinical and pathological parameters determined at the time of biopsy, only glomerular density was significantly associated with subsequent changes in eGFR [5].

The NEPTUNE consortium is a longitudinal, observational cohort study of primary proteinuric glomerular diseases in patients enrolled initially from 21 centers in the United States and Canada [6]. A baseline diagnostic kidney biopsy is required for enrollment. These biopsies were digitized for analysis using a novel digital pathology protocol [7]. Along with quantitative “descriptors” specified by the pathology protocol, additional morphometric parameters were also measured in some biopsies; including average glomerular tuft cross sectional area, cortical glomerular density, and a composite variable including the fractional areas of six cortical compartments (interstitium, intact tubules, atrophic tubules, blood vessels, patent glomeruli, sclerotic glomeruli). Such data provided a unique opportunity to compare combinations of structural variables (along with a complement of 13 demographic and clinical confounding variables) in longitudinal statistical models predicting changes in eGFR over up to 55 months of follow-up.

Our results demonstrated the strong predictive power of quantitative structural parameters assessed on digital images of baseline kidney biopsies of patients enrolled in the NEPTUNE study. With the expanded use of whole slide imaging (WSI) and digital archiving of biopsies, the clinical utility of morphometric analysis on kidney biopsies should be further explored both in the NEPTUNE consortium and other datasets.

## Materials and Methods

We used generalized estimating equations to build a longitudinal prediction model allowing us to investigate the utility of quantitative structural parameters measured on baseline kidney biopsies to predict changes in kidney function in a longitudinal, observational cohort study (NEPTUNE) of primary proteinuric kidney diseases [6]. The outcome of interest was estimated glomerular filtration rate (eGFR). By study design, a minimum follow-up of 30 months was set. Pediatric status was defined as age under 18 years. Estimated GFR was derived from the CKD-EPI equation in adults [8] and from the CKiD equation in children [9].

This study was approved by the IRBs of the 21 individual enrolling academic centers and by the National Institutes of Health. The IRB approval number from the lead center (University of Michigan) is HUM00026609. The approving IRBs were those of the University of Michigan Medical Center, New York University Medical Center, Johns Hopkins Medical Institute, University of Illinois at Chicago, John H Stroger Cook County Hospital, University Health Network–Toronto, Case Western Reserve University, Children's Hospital Los Angeles, Harbor UCLA Biomedical Research Institute, Cohen Children's Hospital, Mayo Clinic (Rochester), Montefiore Medical Center at Albert Einstein University, University of Miami Miller School of Medicine, University of North Carolina at Chapel Hill, Children's Hospital of Pennsylvania with reciprocal agreement for University of Pennsylvania, Seattle Children's Hospital with reciprocal agreement for University of Washington, NIDDK Intramural, Columbia University, Temple University, Emory University, Stanford University, University of Texas at Southwestern. All participants (or for minors, their parents) gave written consent to participate in the NEPTUNE study which included participation in all approved sub-studies. The consent procedure and consent forms were approved by the IRB at each enrolling center. Patients were enrolled from April 2010 to June 2014.

Research subjects were recruited from the NEPTUNE consortium [6], a multicenter longitudinal, observational cohort study of primary proteinuric glomerular diseases in adult and pediatric patients enrolled initially at 21 academic centers in the US and Canada at the time of a clinically-indicated diagnostic kidney biopsy. Requirements for enrollment in the study included a kidney biopsy consistent with minimal change disease (MCD), focal segmental glomerular sclerosis (FSGS) or membranous nephropathy (MN) and quantitative proteinuria greater than 500 mg per 24 hours (or the equivalent value for a ‘spot’ urine protein/creatinine ratio) within 3 months of screening. The study’s renal pathologists made the final qualifying diagnosis after review of the biopsy images. Per NEPTUNE protocol, eGFR was calculated repeatedly every 4 to 6 months during follow-up. Choice of treatment medication was at the discretion of the physicians from the local participating centers.

### Preparation and interpretation of biopsy material

Renal biopsies were obtained by Centers participating in the NEPTUNE study and processed in the usual manner for light, immunofluorescence and electron microscopy. For most centers, de-identified glass slides labeled with the stain and level of sectioning were sent to the National Institutes of Health/National Cancer Institute for central scanning at 40× magnification using an Aperio CS scanning system (Leica MicroSystems, Vista CA). Images were then uploaded on a SlidePath server (Leica MicroSystems, Dublin). Fewer centers performed local scanning using the same protocol and magnification. De-identified scanned whole slide images (WSI), digital images of immunofluorescence slides and electron micrographs, and pathology reports were stored under password-protected access in the web-based NEPTUNE digital pathology repository [7]. Study pathologists reviewed the biopsy materials and assigned cases to the appropriate diagnostic category. The Neptune Digital Pathology Protocol includes evaluation of 60 light microscopic histologic lesions and features, termed descriptors, performed on annotated WSI without morphometry. The descriptors used as structural predictors in this analysis were the estimated extent of interstitial fibrosis (IF) and of tubular atrophy (TA) as these have been identified as significant predictors before and corresponded closely to two of the measured morphometric parameters. These were visually estimated on at least four levels with all available stains (hematoxylin & eosin, trichrome, periodic acid-Schiff, silver methenamine) to increments of 5%. Six renal pathologists estimated IF and TA for each case and the final value was the average of the individual pathologists’ values, as there was excellent concordance on these measures among study pathologists [10].

### Morphometric analysis

**Fig 1:**
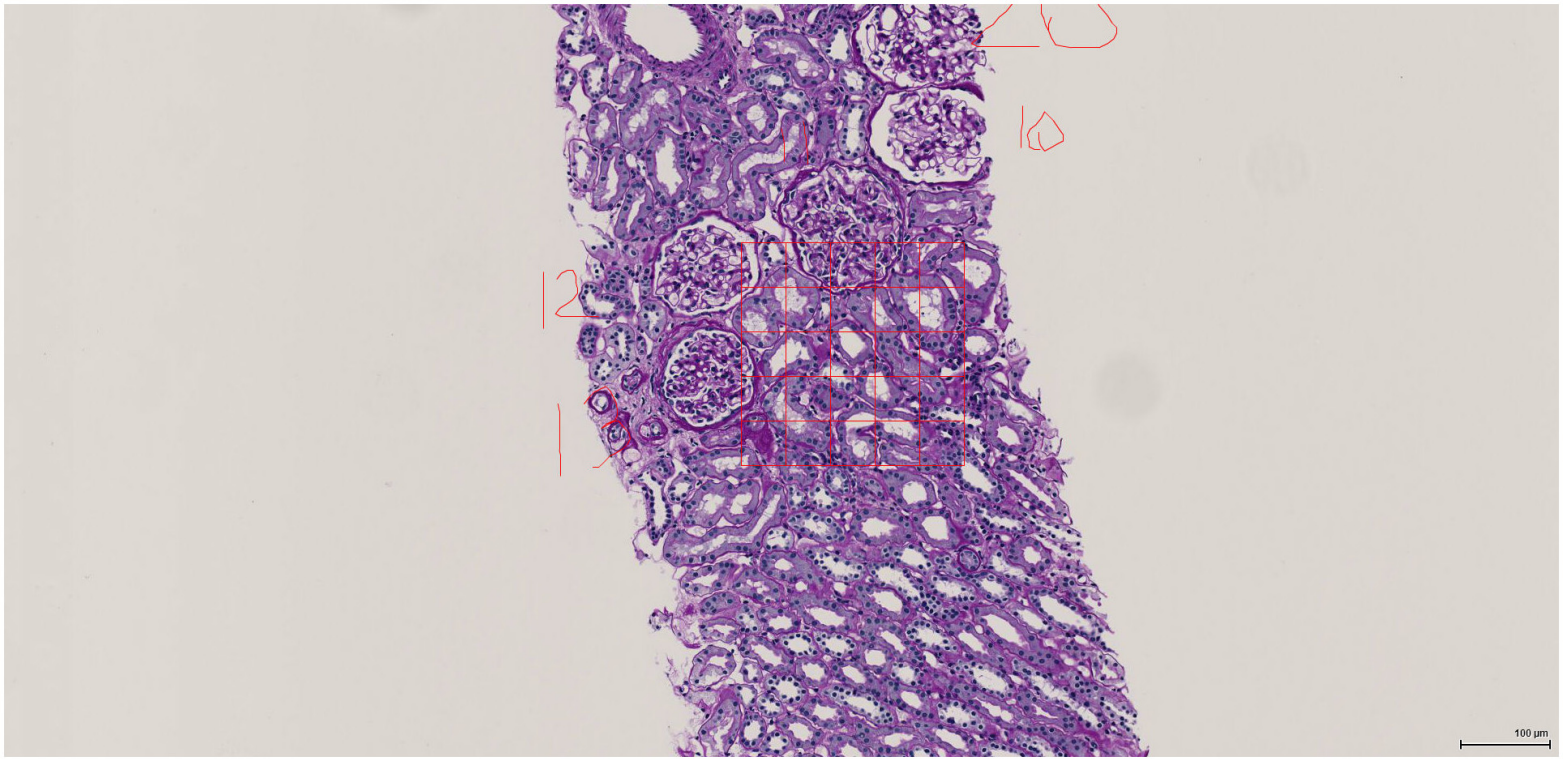
Illustration of the point counting method on a section of cortex (PAS stain). Illustration of the point counting principle for assessing cortical compartments using SlidePath software. The hand-written numbers (10, 11, 12, 13 and part of 20) were added by the slide annotator, to keep track of individual glomeruli. Cross points of the red grid box were used other than the upper and right-side lines (equivalently, using the bottom-left corner point of each grid sub-box). Starting at the top-left, the first point ‘hits’ an intact glomerulus (#12). Moving right, the second point hits an intact tubule. The third hits an intact glomerulus (#11). Twenty-five points per grid are evaluated.

Measurements were made on annotated slides using the SlidePath platform by a single individual (KVL), who was blind to any clinical information on the chosen subjects. Average glomerular tuft area (A_G_) was determined in cases with at least 4 glomeruli represented. Tuft profiles were chosen randomly (uniform random generator) from all the levels in which a particular glomerulus was sectioned. A built-in planimetry program in SlidePath was used for all area measurements. Cortical glomerular density was determined in a single section–usually one near the middle of the stack of slides, usually with PAS staining. The number of patent glomerular tufts in the section was counted and was divided by the cortical area determined by planimetry to give the areal glomerular density, N_A_. Patency was defined by less than two-thirds of the capillary lumens being obliterated. Fractional interstitial area (FIA) and the other fractional cortical areas were measured by imposing 5×5 grids in five different places over the cortex. The definition of the structure on which the 25 points of the grid fell (excluding the upper and right lines) was either interstitium (including extracellular matrix and cells), blood vessels (capillaries, veins/venules, arteries, arterioles), intact tubules, atrophic tubules, patent glomeruli, or globally sclerotic glomeruli. Point counting was done on one section each stained with PAS and with Masson’s trichrome stain (Fig 1). A total of 125 points per stained section were counted, unless the amount of tissue available was insufficient for five distinct cortical locations to be chosen. The morphometric variables used in the longitudinal predictive models were chosen based on their predictive capacity in earlier studies [2–5].

The cortical area glomerular density (N_A_) was used to estimate the cortical volumetric glomerular density (N_V_) using the Wicksell equation: N_V_ = N_A_/D, wherein D is the mean caliper diameter of the glomeruli [11]. The diameter was derived from the average tuft cross-sectional area, $D\;=\;\sqrt{4∙A_{G}/\pi }$, assuming a spherical form of the tuft.

### Statistical analysis

We have tried to adhere to the STROBE reporting guidelines [12] for observational cohort studies. Descriptive statistics included mean ± standard deviation for normally distributed variables and median (IQR) for others. Longitudinal eGFR data was analyzed using the method of generalized estimating equations (GEE); longitudinal prediction models were compared using the quasi-likelihood under independence model criterion (QIC). The QIC is an approximation to the Akaike information criterion (AIC) specifically developed for the GEE approach [13]. QIC calculated in SAS software ignores the autocorrelation of longitudinal outcomes, and some caution is required in its use and interpretation. Internal validation of the models in terms of prediction error was based on leave-one-out cross-validation. The predictive or cross-validated R^2^ is calculated as $R_{pred}^{2}\;=\;1\;–\;PRESS/SSR$, where PRESS is the averaged leave-one-out prediction error to the fitted model and SSR is the averaged sum of squared residuals of modeling.

Thirteen potentially confounding clinical and demographic covariates were included in the predictor sets along with the morphometric parameters: baseline eGFR, race (3 categories), age (at baseline visit), gender, age at onset of proteinuria, duration of disease, diagnostic cohort (2 categories, with membranous nephropathy serving as the reference diagnosis), body mass index and baseline quantitative proteinuria. These covariates were selected in the regression analysis from a larger set of potential confounders by backward elimination. Time of follow-up was included as a covariate to allow for variable lengths of follow-up. Complete data used in the longitudinal models is available (S1 Table).

To choose a parsimonious subset of morphometric parameters, N_A_, N_V_ and A_G_ were included in models along with the first 3 principal components (PC) whose loadings (see below) were derived from the correlation matrix of the six fractional cortical compartments; the models were analyzed by least-angle regression (LAR) on the average rate of change in eGFR [14]. The interpretation of the main component loadings seemed compatible with the order of entry of the principal components into the LAR analysis (PC1 > PC3 > PC2):

PC1 = 0.500 ×FIA+0.548 ×FATA-0.597 ×FITA

PC2 = 0.754 ×FBVA+0.387 ×FSGA+0.374 ×FPGA

PC3 = 0.768 ×FPGA-0.502 ×FSGA-0.305 ×FIA

Wherein the following abbreviations are used: FIA, fractional interstitial area; FATA, fractional atrophic tubule area; FITA, fractional intact tubule area; FBVA, fractional blood vessel area; FSGA, fractional sclerotic glomerular area; FPGA, fractional patent glomerular area. The data analysis was performed using PROC GENMOD in SAS 9.3 for GEE and R software for LAR.

## Results

**Fig 2:**
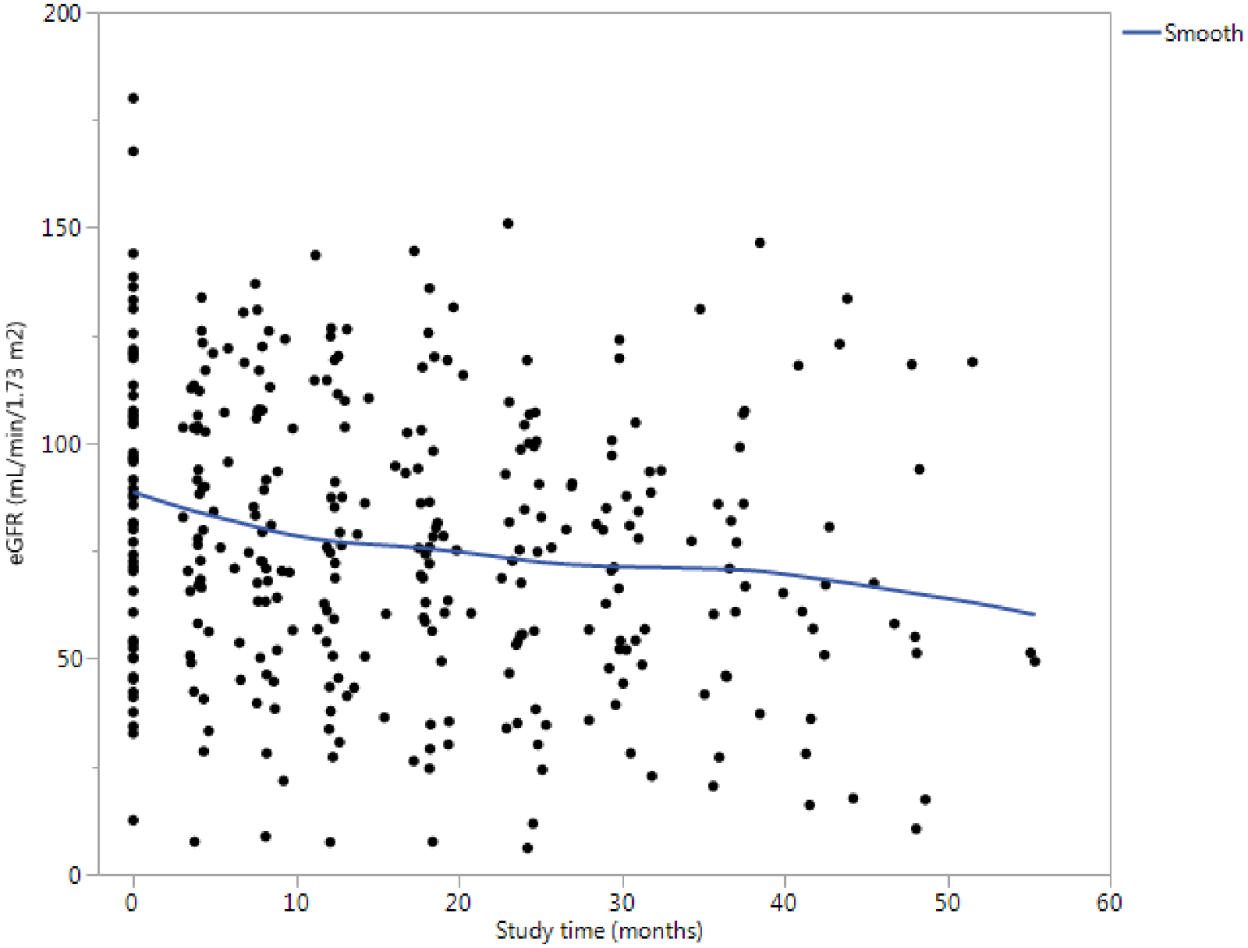
Time-course of change in eGFR over follow-up. All eGFR values used in the analysis are plotted and a smoothed curve fitted to the entire cohort to reflect overall time-related change in eGFR. The eGFR is fit to the study times using kernel density estimation (with λ = 0.77). The linear fit slope is -3.36 mL/min/1.73m^2^/year.

The outcome variable in the longitudinal predictive modeling was the change in eGFR during follow-up ranging from 0 months to 55.4 months (median 30.4 months). The inclusion of 7 individuals with only a baseline eGFR was useful to better estimate baseline cohort effects as part of the longitudinal models. The median initial eGFR was 89.5 (IQR = 64.5–108.4) mL/min/1.73m^2^. The average rate of change in eGFR over follow-up was -3.36 mL/min/1.73m^2^/year (Fig 2). One of the patients in the cohort progressed to ESRD before the second study visit and was excluded. All available eGFR values were used in the statistical analysis (a median of 2 repeated measures per year of follow-up).

Measurements were made of three classes of morphometric variables on the digital images of the baseline biopsies in 83 subjects (average glomerular tuft area, A_G_; areal cortical density of glomeruli, N_A_; and the fractional areas of six cortical compartments, detailed below). For most of these subjects, both a complete morphometric analysis and a complete evaluation using the NEPTUNE digital pathology protocol [7] were possible. The digital pathology protocol includes descriptors for the estimated percentages of interstitial fibrosis and of tubular atrophy, categories analogous to two of the morphometric fractional areas. A subset of 56 subjects having complete morphometric and digital pathology evaluations, as well as complete observations of 13 confounding variables, was deemed adequate for analysis. Individuals were excluded because of missing data at the baseline visit, development of end-stage kidney failure before scheduled follow-up, an off-target biopsy diagnosis and the existence of a prior biopsy. Of the 56 subjects with complete data, 25% were African-American, 70% were male and 39% were children; 25 had FSGS, 19 had minimal change disease, and 12 had membranous nephropathy.

Tuft cross-sectional area was measurable in at least four glomerular cross sections in 83 cases, with an average of 23±14 SD (range 4–75) uniquely identified glomeruli per case. The six fractional areas (interstitium, intact tubules, atrophic tubules, blood vessels, patent glomeruli, sclerotic glomeruli) were determined by point-counting within the cortex on both PAS-stained and Trichrome-stained slides. The estimates of the fractional interstitial area, for example, on the PAS- and Trichrome-stained slides from the same biopsy agreed quite well (Pearson’s r = 0.872). Several of the cortical compartment components were significantly correlated with each other (Table 1).

**Table 1:** Pearson correlations among fractional cortical areas.

	FIA	FBVA	FITA	FATA	FPGA	FSGA
FIA	1.000	0.026	**-0.867**	**0.670**	**-0.308**	**0.324**
FBVA	0.026	1.000	-0.213	-0.092	0.044	0.123
FITA	**-0.867**	-0.213	1.000	**-0.852**	0.016	**-0.411**
FATA	**0.670**	-0.092	**-0.852**	1.000	-0.149	0.275
FPGA	**-0.308**	0.044	0.016	-0.149	1.000	-0.097
FSGA	**0.324**	0.123	**-0.411**	0.275	-0.097	1.000

Bold indicates a significant correlation (using the Bonferroni correction for multiple comparisons). FIA, fractional interstitial area; FBVA, fractional blood vessel area; FITA, fractional intact tubule area; FATA, fractional atrophic tubule area; FPGA, fractional patent glomerular area; FSGA, fractional sclerotic glomerular area.

Correlation between two of the morphometric parameters (determined by point counting) and their corresponding pathology descriptors was analyzed by Pearson correlation and the results are summarized in Table 2. The morphometric parameter, fractional interstitial area (FIA), was strongly correlated with its analogous digital pathology descriptor, interstitial fibrosis (IF), estimated by the study pathologists. The morphometric and digital pathology assessments of tubular atrophy were also strongly correlated, both on PAS- and Trichrome-stained slides (Table 2). Interstitial fibrosis (IF) estimated from the same slides by three pathologists in comparison had Pearson’s *r* values from 0.87 to 0.99; estimated tubular atrophy (TA) had *r* values from 0.87 to 0.98 among the pathologists.

**Table 2:** Correlations between morphometric variables (FIA, FATA) and corresponding pathology descriptors (IF, TA).

Pearson correlation (r)	FIA *vs*. IF	FATA *vs*. TA
Trichrome	0.852	0.822
PAS	0.802	0.814

P<0.0001 for all pairwise correlations. n = 77–79. FIA, fractional interstitial area; IF, interstitial fibrosis; FATA, fractional atrophic tubular area; TA, tubular atrophy.

To reduce the dimensionality of the structural parameters, using their correlations we derived the principal components (PC) of the six individual fractional cortical component areas determined by point counting on the PAS-stained slides. We then used the top three principal components as a substitute for the six specific fractional compartment components in some of the predictive models. The principal components are intended to retain the greatest amount of the data variability (essential for prediction) in the smallest number of variables. See Methods for the specific loadings.

In order to identify the strongest morphometric predictors in a parsimonious predictive model, we performed least-angle regression (LAR) on the longitudinal eGFR [3] using the morphometric variables alone as predictors (N_A_, A_G_, N_V_ and the top 3 principal components from the six fractional areas). LAR is a sequential model-selection method that helps to avoid over-fitting in prediction models and more importantly to rank the predictors’ importance by the order of their entry into the model. It is worth noting that the same order of entry into the LAR model occurred for fractional areas measured on either the PAS- or Trichrome-stained slides: PC1 > N_A_ > A_G_ > PC3 > PC2 > N_V_.

As a comparison model containing no structural variables, thirteen demographic and clinical variables were related to the change in eGFR over time using GEE. Of these predictors, only baseline eGFR and MCD diagnosis were significant predictors of change in eGFR (P<0.0001 and P = 0.01). The follow-up time covariate was also significant in the model (P = 0.001), but this was a default covariate included only to adjust for the longitudinally measured eGFR and was not considered to be a clinical predictor variable.

Four different combinations of four of the nine digital pathology and morphometric parameters were analyzed by GEE, adjusting for the 13 clinical predictor variables (Table 3). Mean glomerular tuft area (A_G_) and cortical glomerular density (N_A_) were used in all of the models containing structural predictors. The remaining structural predictors were added in several ways. Model 1 included the digital pathology descriptors IF and TA. Model 2 included the morphometric parameters, FIA and FATA, which are analogues of IF and TA. Model 3 included the first two principal components of the cortical components (PC1, PC2). Model 4 included the principal components PC1 and PC3, since this reflected the order of the principal components in the LAR analysis.

**Table 3:** Four generalized estimating equation models based on 13 baseline clinical/demographic and 4 structural variables per model. Pr(>|W|) represents the Wald statistic of the model parameter.

Model 1	Pr(>|W|)	Model 2	Pr(>|W|)
eGFR	**<0.0001**	eGFR	**<0.0001**
Race: Asian	0.518	Race: Asian	0.667
Race: Black	0.955	Race: Black	0.725
Race: White	0.852	Race: White	0.606
Patient age	0.499	Patient age	0.247
Female	0.180	Female	0.105
Patient age at onset of disease	0.489	Patient age at onset of disease	0.241
Duration of disease	0.553	Duration of disease	0.238
Patient Cohort–MCD	**0.014**	Patient Cohort–MCD	**0.012**
Patient Cohort–FSGS	0.242	Patient Cohort–FSGS	0.407
BMI	0.317	BMI	0.322
Urine protein/creatinine ratio	**0.023**	Urine protein/creatinine ratio	0.240
Time of follow-up (months)	0.001	Time of follow-up (months)	0.001
Interstitial Fibrosis	0.403	FIA (PAS)	0.932
Tubular Atrophy	0.688	FATA (PAS)	**0.0004**
Mean Glomerular Tuft Area	0.630	Mean Glomerular Tuft Area	0.471
Cortical Density of Patent Glomeruli	0.526	Cortical Density of Patent Glomeruli	0.416
QIC	409.502	QIC	409.53
R^2^_pred_	62.6%	R^2^_pred_	61.9%
Sample size	56	Sample size	56
Model 3	Pr(>|W|)	Model 4	Pr(>|W|)
eGFR	**<0.0001**	eGFR	**<0.0001**
Race: Asian	0.499	Race: Asian	0.736
Race: Black	0.798	Race: Black	0.659
Race: White	0.756	Race: White	0.538
Patient age	0.115	Patient age	0.347
Female	0.098	Female	0.100
Patient age at onset of disease	0.111	Patient age at onset of disease	0.338
Duration of disease	0.114	Duration of disease	0.326
Patient Cohort–MCD	**0.013**	Patient Cohort–MCD	**0.021**
Patient Cohort–FSGS	0.406	Patient Cohort–FSGS	0.472
BMI	0.326	BMI	0.349
Urine protein/creatinine ratio	0.128	Urine protein/creatinine ratio	0.184
Time of follow-up (months)	0.001	Time of follow-up (months)	0.001
PC1	**0.005**	PC1	**0.009**
PC2	**0.041**	PC3	0.612
Mean Glomerular Tuft Area	0.510	Mean Glomerular Tuft Area	0.449
Cortical Density of Patent Glomeruli	0.337	Cortical Density of Patent Glomeruli	0.453
QIC	407.83	QIC	410.63
R^2^_pred_	62%	R^2^_pred_	61.3%
Sample size	56	Sample size	56

IF, interstitial fibrosis; TA, tubular atrophy; PC1, first principal component of 6 cortical compartments; PC2, second principal component of 6 cortical compartments; PC3, third principal components of 6 cortical compartments; FIA, fractional interstitial area; FATA, fraction atrophic tubular area. QIC is an abbreviation of Quasi Information Criterion, which is used to characterize the goodness-of-fit of data to a longitudinal GEE model. The smaller the QIC value, the better the model fit. R^2^_pred_ designates the predictive (or cross-validated) coefficient of determination. Bold type indicates significant contribution of a factor to the GEE.

The quality of the model fit was assessed by the quasi-information criterion (QIC) as part of the standard SAS output. The QIC was lowest (preferred) in Model 3, followed by Models 1 and 2, although all four models were quite similar in this regard. The estimated prediction capacity was determined, through the method of leave-one-out cross-validation, by the percent of predicted residual sum of squares (PRESS), R^2^_pred_, for a candidate model relative to the default model with only the time covariate. The estimated prediction capacities of the four models were quite similar, between 61.3 and 62.6%.

Interestingly, one clinical variable (baseline quantitative proteinuria) that was not a significant contributor to the GEE in the clinical predictor-only model turned out to be a significant predictor (P = 0.02) in one of the four models that included structural predictors (Model 1).

**Fig 3:**
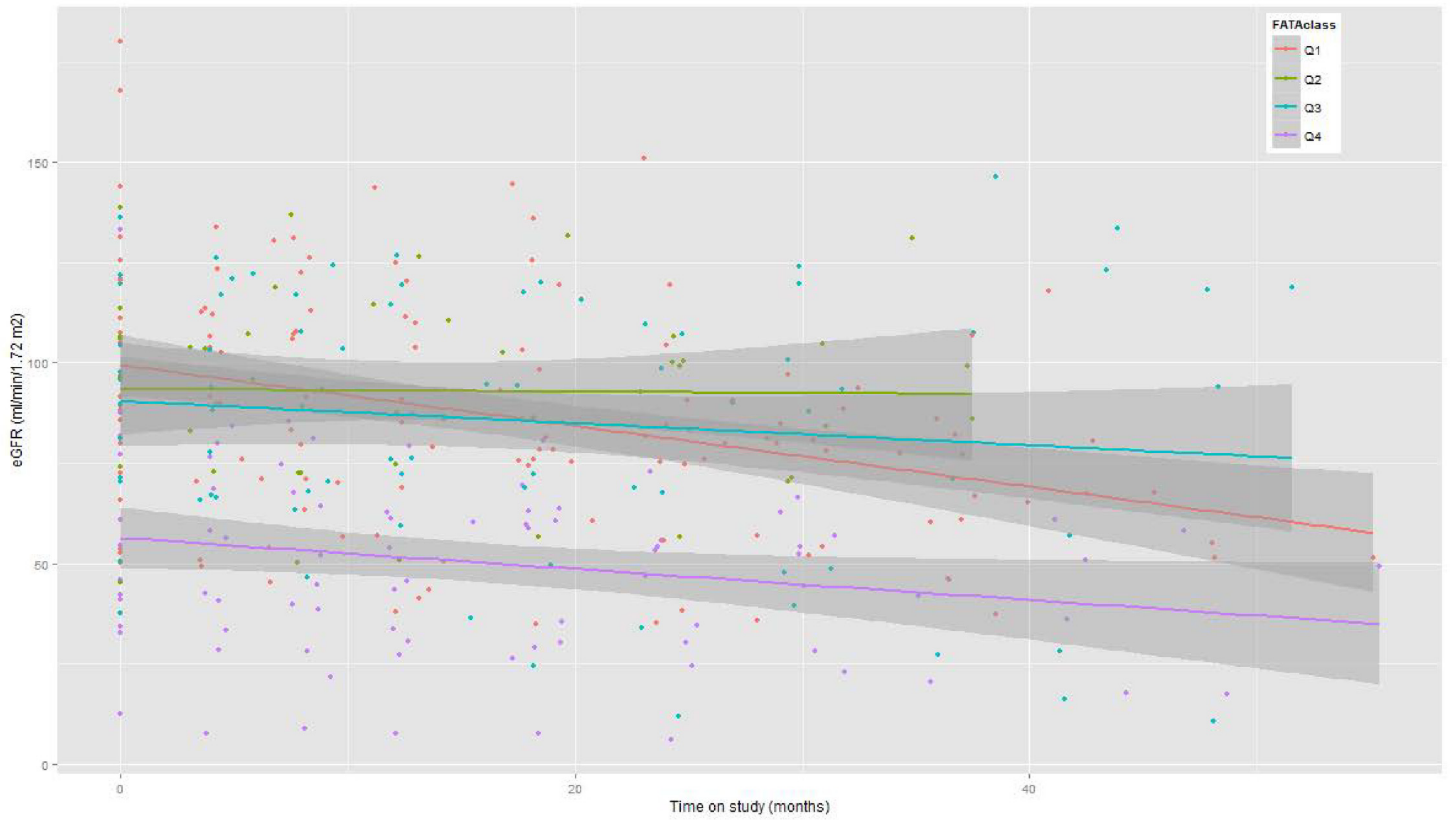
Observed eGFR progression patterns over follow-up stratified by quartiles of FATA. Linear fits to observed eGFR values stratified by FATA from lowest (red) to highest (purple). Initial eGFR is highest for the lowest FATA quartile and decreases with each quartile. Compared to the reference category of the lowest FATA quartile, testing for the differential eGFR slopes of the second, third and fourth quartiles yielded P values of 0.15, 0.25 and <0.001, respectively. The apparent more negative slope of the lowest quartile may be due to a smaller number of long follow-up points.

In the three models including morphometric variables (Models 2–4), one or more of the morphometric variables made a significant contribution to each model. The fractional atrophic tubule area (FATA) had a quite strong effect (Fig 3). Baseline eGFR and MCD diagnosis made significant contributions to each model. In general, the contributions of the morphometric variables were stronger than any predictor variable other than baseline eGFR.

## Discussion

In 56 subjects among the longitudinal NEPTUNE cohort of pediatric and adult patients with primary proteinuric glomerular disease, structural variables measured in digital images of baseline kidney biopsies were among the strongest predictors of the change in eGFR over their first 55 months of follow-up. This was the case for several combinations of different structural variables. Only baseline eGFR was found to be a stronger predictor among the 13 demographic and clinical confounding covariates. Inclusion of structural variables in longitudinal statistical models predicting changes in eGFR in such diseases therefore seems justified.

Although quantitative structural parameters measured on kidney biopsy have been found before to be strong predictors of clinical outcome [2–5], they have generally not been included in multivariate statistical models predicting CKD progression [1]. When structural parameters have been included in such models, it has usually been as qualitative or semi-quantitative (categorical) variables.

Even in baseline biopsies, presumably reflecting early disease, there may be significant variation in morphometric parameters, a feature necessary for potential utility in prediction. A four-to-seven-fold variation in cortical glomerular density, for example, has been reported in groups of patients with different renal diseases [4, 5]. Glomerular density has also been measured in intraoperative biopsies of over 1000 living kidney donors, presumed to be free of diabetes, hypertension or proteinuria [15]. The variation in density was less than in patients with glomerular disease (a 35% coefficient of variation in glomerular density) suggesting the influence of glomerulopenia on disease susceptibility or the presence of glomerular loss even in baseline biopsies. Glomerular density was also inversely related to glomerular tuft area [15], suggesting its relevance as a marker of total glomerular number.

Some of the structural variables examined in this study were chosen because of their significance in other predictive studies of glomerular disease [2–5]. They may in general be considered to represent acquired nephron loss (FIA/IF, FATA/TA) or congenital nephron endowment (N_A_). Fractional interstitial area (a quantitative measure of interstitial fibrosis) would be expected to increase with disease-related nephron loss. The cortical density of glomerular profiles (N_A_), on the other hand, could represent congenital nephron endowment, the effects of glomerular sclerosis and tuft resorption with disease, or both. Average glomerular tuft area (A_G_) increases as a result of compensatory glomerular hypertrophy after nephron loss, but has also been found to be related to inborn nephron number and cortical glomerular density [15,16]. The strong collinearity among structural variables may explain the lack of significant contributions of more of them individually to the multivariate predictive models, which was a rationale for using principal components as combined predictors.

The lowest QIC of the GEE models (representing the ‘best’ model fit) was for model 3. QIC calculated in SAS software does not account for the temporal correlation of longitudinal eGFR measures, and hence should be used with caution [13, 17]. The statistical significance of the structural variables generally exceeded that of any of the clinical variables other than baseline eGFR and argues for their inclusion in future clinical predictive models. The estimated predictive capacity of the various models was quite similar probably due to the effects of a few stronger predictors in the models. The performance of the structural variables in the predictive models is more impressive considering the heterogeneity of the subjects, including both children and adults and including several different disease diagnoses.

Although the pathology descriptors (IF/TA) and their morphometric analogues (FIA, FATA) were strongly correlated, only the morphometric predictor variables made significant contributions to the longitudinal eGFR models. This surprising finding and the reasons for it need to be validated in further studies directly comparing the two approaches. This study was limited by the sample size based on subjects who had complete baseline data, digital pathology, morphometry and eGFR follow-up. Although quantitative structural variables made strong contributions to the predictive models in the context of standard clinical and demographic variables, it is distinctly possible that even stronger predictive models would result from combining morphometric variables with novel biomarkers, such as fibroblast growth factor 23 (FGF23) or soluble urokinase-type plasminogen activator receptor (suPAR) [18].

Large, complex cohort studies such as NEPTUNE are costly and resource-intensive. Risk stratification of potential subjects therefore may play a critical role in the efficient design and execution of such studies. We propose that quantitative structural parameters, particularly morphometric parameters such as FIA and FATA, be routinely included in risk stratification models for any such studies that include a renal biopsy. At the same time, measurement of the complete set of purely morphometric parameters in the current study was very time-consuming. Even if these morphometric parameters prove to be powerful contributors to predictive models of change in GFR, it is unlikely that measuring them in kidney biopsies would ever be incorporated into routine clinical practice on the basis of the current approach. Given the increasing use of WSI and digital pathology itself, however, it is possible that automated or semi-automated systems can be designed that might capture much of the same morphometric information as FIA or FATA, perhaps using specific tissue stains [19, 20]. If structural information with the same prognostic significance could be determined from digital biopsy images by automated methods, such parameters might someday become part of routine renal pathology evaluation and might help to assess the risk of progressive loss of renal function in individuals presenting with primary proteinuric glomerular disease.

## Supporting Information

S1 Table Morphometric, clinical and demographic data used in generalized estimating equation modeling. (CSV)

## References

[1] Tangri N, Kitsios GD, Inker LA, Griffith J, Naimark DM, Walker S, et al Risk prediction models for patients with chronic kidney disease. *Ann Intern Med* 2013; 158:596–603. 10.7326/0003-4819-158-8-201304160-00004 23588748

[2] Bohle A, Mackensen-Haen S, von Gise H, Grund KE, Wehrmann M, Batz C, et al The consequences of tubulo-interstitial changes for renal function in glomerulopathies. A morphometric and cytological analysis. *Path Res Pract* 1990; 186:135–144. 2315207 10.1016/S0344-0338(11)81021-6

[3] Lemley KV, Lafayette RA, Derby G, Blouch KL, Anderson L, Efron B, et al Prediction of early progression in recently diagnosed IgA nephropathy. *Nephrol Dial Transplant* 2008; 23:213–222. 17890749 10.1093/ndt/gfm560

[4] Tsuboi N, Kawamura T, Koike K, Okonogi H, Hirano K, Hamaguchi A, et al Glomerular density in renal biopsy specimens predicts the long-term prognosis of IgA nephropathy. *Clin J Am Soc Nephrol* 2010; 5:39–44. 10.2215/CJN.04680709 19965542 PMC2801658

[5] Tsuboi N, Kawamura T, Miyazaki Y, Utsunomiya Y, Hosoya T. Low glomerular density is a risk factor for progression in idiopathic membranous nephropathy. *Nephrol Dial Transplant* 2011; 26:3555–3560. 10.1093/ndt/gfr399 21771759

[6] Gadebeku C, Gipson DS, Holzman LB, Ojo AO, Song PX, Barisoni L, et al Design of the nephrotic syndrome study network (NEPTUNE) to evaluate primary glomerular nephropathy by a multidisciplinary approach. *Kidney Int* 2013; 83:749–756. 10.1038/ki.2012.428 23325076 PMC3612359

[7] Barisoni L, Nast CC, Jennette JC, Hodgin JB, Herzenberg AM, Lemley KV, et al Digital pathology evaluation in the multicenter nephrotic syndrome study network (NEPTUNE). *Clin J Am Soc Nephrol* 2013; 8:1449–1459. 10.2215/CJN.08370812 23393107 PMC3731905

[8] Levey AS, Stevens LA, Schmid CH, Zhang YL, Castro AF 3rd, Feldman HI, et al A new equation to estimate glomerular filtration rate. *Ann Intern Med* 2009; 150:604–612. 19414839 10.7326/0003-4819-150-9-200905050-00006 PMC2763564

[9] Schwartz GJ, Munoz A, Schneider MF, Mak RH, Kaskel F, Warady BA, et al New equations to estimate GFR in children with CKD. *J Am Soc Nephrol* 2009; 20:629–637. 10.1681/ASN.2008030287 19158356 PMC2653687

[10] Barisoni L, Troost JP, Nast C, Bagnasco S, Avila-Casado C, Hodgin J, et al Reproducibility of the NEPTUNE descriptor-based scoring system on whole-slide images and histologic and ultrastructural digital images. Mod Pathol; 10.1038/modpathol.2016.58 PMC5515468 27102348

[11] Lemley KV, Bertram JF, Nicholas SB, White K. Estimation of glomerular podocyte number: a selection of valid methods. *J Am Soc Nephrol* 2013; 24:1193–1202. 10.1681/ASN.2012111078 23833256 PMC3736716

[12] von Elm E, Altman DG, Egger M, Pocock SJ, Gøtzsche PC, Vandenbroucke JP for the STROBE Initiative. The strengthening the reporting of observational studies in epidemiology (STROBE) statement: guidelines for reporting observational studies. *J Clin Epidemiol* 2008; 61:344–349. 10.1016/j.jclinepi.2007.11.008 18313558

[13] Pan W. Akaike’s information criterion in generalized estimating equations. *Biometrics* 2001; 57:120–125. 11252586 10.1111/j.0006-341x.2001.00120.x

[14] Efron B, Hastie T, Johnstone I, Tibshirani R. Least angle regression. *Ann Stat* 2004; 32:407–499.

[15] Rule AD, Semret MH, Amer H, Cornell LD, Taler SJ, Lieske JC, et al Association of kidney function and metabolic risk factors with density of glomeruli on renal biopsy samples from living donors. *Mayo Clin Proc* 2011; 86:282–290. 10.4065/mcp.2010.0821 21454731 PMC3068887

[16] Puelles VG, Douglas-Denton RN, Zimanyi MA, Armitage JA, Hughson MD, Kerr PG, et al Glomerular hypertrophy in subjects with low nephron number: contributions of sex, body size and race. *Nephrol Dial Transplant* 2014; 29:1686–1695. 10.1093/ndt/gfu088 24792374 PMC4145866

[17] Song PXK, Jiang Z, Park E, Qu A. Quadratic inference functions in marginal models for longitudinal data. *Statist Med* (2009); 28:3683–3696. 10.1002/sim.3719 19757486

[18] Hayek SS, Sever S, Ko YA, Trachtman H, Awad M, Wadhwani S, et al Soluble urokinase receptor and chronic kidney disease. *New Engl J Med* 2015; 373:1916–1925. 10.1056/NEJMoa1506362 26539835 PMC4701036

[19] Servais A, Meas-Yedid V, Noël LH, Martinez F, Panterne C, Kreis H, et al Interstitial fibrosis evolution on early sequential screening renal allograft biopsies during quantitative image analysis. *Am J Transplant* 2011; 11:1456–1463. 10.1111/j.1600-6143.2011.03594.x 21672152

[20] Farris AB, Chan S, Climenhaga J, Adam B, Bellamy CO, Serón D, et al Banff fibrosis study: multicenter visual assessment and computerized analysis of interstitial fibrosis in kidney biopsies. *Am J Transplant* 2014; 14:897–907. 10.1111/ajt.12641 24712330

